# 4-Carboxy­pyridazin-1-ium chloride

**DOI:** 10.1107/S1600536808022319

**Published:** 2008-07-19

**Authors:** Wojciech Starosta, Janusz Leciejewicz

**Affiliations:** a Institute of Nuclear Chemistry and Technology, ulica Dorodna 16, 03-195 Warszawa, Poland

## Abstract

The structure of the title compound, C_5_H_5_N_2_O_2_^+^·Cl^−^, is composed of chloride anions and 4-carboxy­pyridazin-1-ium cations. Chloride anions bridge the cations *via* O—H⋯Cl and N—H⋯Cl hydrogen bonds to form ribbons. The latter, linked by van der Waals forces with lengths in the range 3.254 (2)–3.497 (2) Å, form coplanar layers. Very weak inter­actions operate also between adjacent layers, as indicated by their spacing of 3.339 (1) Å.

## Related literature

For the crystal structure of pyridazine-3-carboxylic acid hydro­chloride, see: Gryz *et al.* (2003 ▶). For a report of mol­ecular layers in the structure of pyrazine-2-carboxylic acid, see: Takusagawa *et al.* (1974 ▶).
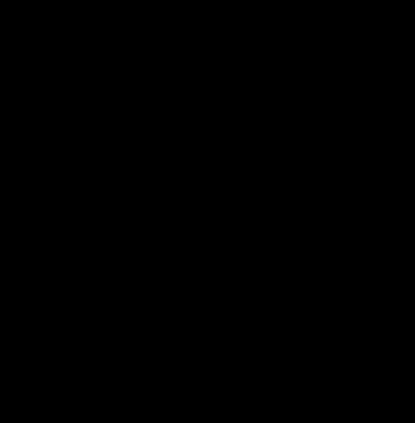


## Experimental

### 

#### Crystal data

C_5_H_5_N_2_O_2_^+^·Cl^−^
*M* *_r_* = 160.56
Monoclinic, 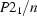

*a* = 6.8505 (14) Å
*b* = 6.5905 (13) Å
*c* = 14.561 (3) Å
β = 97.65 (3)°
*V* = 651.6 (2) Å^3^
*Z* = 4
Mo *K*α radiation
μ = 0.52 mm^−1^
*T* = 293 (2) K
0.39 × 0.16 × 0.12 mm

#### Data collection

Kuma KM-4 four-circle diffractometer
Absorption correction: analytical (*CrysAlis RED*; Oxford Diffraction, 2008 ▶) *T*_min_ = 0.942, *T*_max_ = 0.952
2062 measured reflections
1917 independent reflections
1318 reflections with *I* > 2σ(*I*)
*R*_int_ = 0.024
3 standard reflections every 200 reflections intensity decay: 1.1%

#### Refinement

*R*[*F* ^2^ > 2σ(*F* ^2^)] = 0.030
*wR*(*F* ^2^) = 0.104
*S* = 1.03
1917 reflections
99 parameters
H atoms treated by a mixture of independent and constrained refinement
Δρ_max_ = 0.35 e Å^−3^
Δρ_min_ = −0.21 e Å^−3^

### 

Data collection: *KM-4 Software* (Kuma, 1996 ▶); cell refinement: *KM-4 Software*; data reduction: *DATAPROC* (Kuma, 2001 ▶); program(s) used to solve structure: *SHELXS97* (Sheldrick, 2008 ▶); program(s) used to refine structure: *SHELXL97* (Sheldrick, 2008 ▶); molecular graphics: *SHELXTL* (Sheldrick, 2008 ▶); software used to prepare material for publication: *SHELXL97*.

## Supplementary Material

Crystal structure: contains datablocks I, global. DOI: 10.1107/S1600536808022319/rk2101sup1.cif

Structure factors: contains datablocks I. DOI: 10.1107/S1600536808022319/rk2101Isup2.hkl

Additional supplementary materials:  crystallographic information; 3D view; checkCIF report

## Figures and Tables

**Table 1:** Hydrogen-bond geometry (Å, °)

*D*—H⋯*A*	*D*—H	H⋯*A*	*D*⋯*A*	*D*—H⋯*A*
O2—H2⋯Cl1	0.91 (3)	2.05 (3)	2.9464 (14)	169 (2)
N1—H1⋯Cl1^i^	0.92 (3)	2.15 (3)	3.0373 (15)	160 (2)

Symmetry code: (i) 
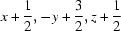
.

**Fig. 1.**
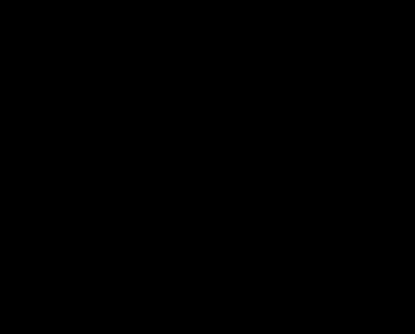
A molecular structure of I with the atom labelling scheme. The displacement ellipsoids are drawn at 50% probability level. H atoms are presented as a small spheres of arbitrary radius.

**Fig. 2.**
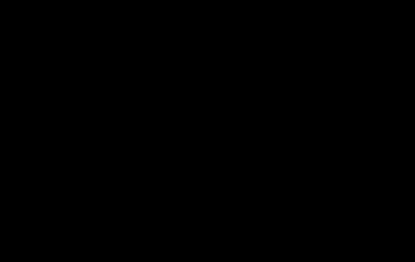
The structure packing diagram of I.

## supplementary crystallographic information

### Comment

The structure of the title compound (C_5_H_5_N_2_O_2_)^+^ Cl^-^, I, is built from chloride anions and heterocycle cations. Chloride anions bridge the cations *via* hydrogen bonds O2—H2···Cl1 2.05 (3)Å and N1—H1···Cl1^i^ 2.15 (3)Å to form ribbons; symmetry code: (i) x+1/2, -y+3/2, z+1/2. The ribbons linked by van der Waals forces with lengths in the range from 3.254 (2) to 3.497 (2)Å make coplanar layers. The shortest distance between pyridazine rings belonging to adjacent layers is 3.339 (1)Å. The pyridazine ring are planar (r.m.s. 0.0060Å) and formes with the carboxylate group (C7/O1/O2) dihedral angle 27.7 (1)°. Bond lengths and bond angles within the cation agree well with those reported in the structure of pyridazine–3–carboxylic acid hydrochloride (Gryz *et al.*, 2003).

### Experimental

Single crystals of I were obtained by recrystallization of pyridazine–4–carboxylic acid (ALDRICH) from warm 1*M* solution of hydrochloric acid. Attempts to recrystallize from water and alcohols yielded specimens unsuitable for collecting X–ray data.

### Refinement

All H atoms bonded with C atoms were positioned geometrically and refined in riding model approximation with C—H = 0.93Å and U_iso_(H) = 1.2U_eq_(C). The H atoms connected with N and O atoms were located in difference Fourier map and refined isotropically.

### Crystal data

**Table:**

C_5_H_5_N_2_O_2_^+^·Cl^–^	*F*_000_ = 328
*M**_r_* = 160.56	*D*_x_ = 1.637 Mg m^−^^3^
Monoclinic, *P*2_1_/*n*	Mo *K*α radiation λ = 0.71073 Å
*a* = 6.8505 (14) Å	Cell parameters from 6 reflections
*b* = 6.5905 (13) Å	θ = 6–15º
*c* = 14.561 (3) Å	µ = 0.52 mm^−^^1^
β = 97.65 (3)º	*T* = 293 (2) K
*V* = 651.6 (2) Å^3^	Block, colourless
*Z* = 4	0.39 × 0.16 × 0.12 mm

### Data collection

**Table:**

Kuma KM-4 four-circle diffractometer	*R*_int_ = 0.024
Radiation source: Fine–focus sealed tube	θ_max_ = 30.1º
Monochromator: Graphite	θ_min_ = 2.8º
*T* = 293(2) K	*h* = −9→0
Profile data from ω/2θ scans	*k* = 0→9
Absorption correction: Analytical(CrysAlis RED; Oxford Diffraction, 2008)	*l* = −20→20
*T*_min_ = 0.942, *T*_max_ = 0.952	3 standard reflections
2062 measured reflections	every 200 reflections
1917 independent reflections	intensity decay: 1.2%
1318 reflections with *I* > 2σ(*I*)	

### Refinement

**Table:**

Refinement on *F*^2^	Secondary atom site location: Difmap
Least-squares matrix: Full	Hydrogen site location: Geom
*R*[*F*^2^ > 2σ(*F*^2^)] = 0.030	H atoms treated by a mixture of independent and constrained refinement
*wR*(*F*^2^) = 0.104	*w* = 1/[σ^2^(*F*_o_^2^) + (0.0577*P*)^2^ + 0.1527*P*] where *P* = (*F*_o_^2^ + 2*F*_c_^2^)/3
*S* = 1.03	(Δ/σ)_max_ = 0.001
1917 reflections	Δρ_max_ = 0.36 e Å^−^^3^
99 parameters	Δρ_min_ = −0.21 e Å^−^^3^
Primary atom site location: Direct	Extinction correction: None

### Special details

**Table:**

Geometry. All s.u.'s (except the s.u. in the dihedral angle between two l.s. planes) are estimated using the full covariance matrix. The cell s.u.'s are taken into account individually in the estimation of s.u.'s in distances, angles and torsion angles; correlations between s.u.'s in cell parameters are only used when they are defined by crystal symmetry. An approximate (isotropic) treatment of cell s.u.'s is used for estimating s.u.'s involving l.s. planes.
Refinement. Refinement of *F*^2^ against ALL reflections. The weighted *R*–factor *wR* and goodness of fit *S* are based on *F*^2^, conventional *R*–factors *R* are based on *F*, with *F* set to zero for negative *F*^2^. The threshold expression of *F*^2^ > σ(*F*^2^) is used only for calculating *R*–factors(gt) *etc*. and is not relevant to the choice of reflections for refinement. *R*–factors based on *F*^2^ are statistically about twice as large as those based on *F*, and *R*–factors based on ALL data will be even larger.

### Fractional atomic coordinates and isotropic or equivalent isotropic displacement parameters (Å^2^)

**Table:**

	*x*	*y*	*z*	*U*_iso_*/*U*_eq_	
Cl1	0.61082 (6)	0.18289 (6)	0.39362 (3)	0.03696 (13)	
O1	0.8041 (2)	0.6663 (2)	0.46266 (8)	0.0485 (3)	
C3	0.8870 (3)	0.9337 (3)	0.61752 (11)	0.0382 (4)	
H3	0.8395	0.9943	0.5613	0.046*	
C4	0.8811 (2)	0.7225 (2)	0.62339 (10)	0.0296 (3)	
O2	0.7273 (2)	0.4276 (2)	0.56133 (8)	0.0414 (3)	
C7	0.7988 (2)	0.6028 (3)	0.53974 (10)	0.0330 (3)	
C5	0.9550 (2)	0.6321 (2)	0.70515 (11)	0.0337 (3)	
H5	0.9548	0.4918	0.7120	0.040*	
N2	0.9559 (2)	1.0516 (2)	0.68707 (10)	0.0414 (3)	
C6	1.0306 (3)	0.7589 (3)	0.77771 (11)	0.0376 (4)	
H6	1.0841	0.7045	0.8345	0.045*	
N1	1.0257 (2)	0.9555 (2)	0.76522 (10)	0.0368 (3)	
H1	1.063 (3)	1.042 (4)	0.8143 (17)	0.063 (7)*	
H2	0.675 (4)	0.358 (4)	0.5097 (19)	0.069 (8)*	

### Atomic displacement parameters (Å^2^)

**Table:**

	*U*^11^	*U*^22^	*U*^33^	*U*^12^	*U*^13^	*U*^23^
Cl1	0.0472 (2)	0.02641 (19)	0.0353 (2)	0.00189 (16)	−0.00212 (15)	−0.00355 (14)
O1	0.0698 (9)	0.0472 (8)	0.0264 (6)	0.0011 (7)	−0.0010 (5)	0.0038 (5)
C3	0.0507 (10)	0.0319 (8)	0.0309 (7)	0.0058 (7)	0.0017 (7)	0.0058 (6)
C4	0.0313 (7)	0.0310 (7)	0.0259 (6)	0.0027 (6)	0.0021 (5)	0.0015 (5)
O2	0.0567 (8)	0.0354 (6)	0.0303 (6)	−0.0046 (6)	−0.0010 (5)	−0.0028 (5)
C7	0.0371 (8)	0.0336 (8)	0.0267 (7)	0.0064 (6)	−0.0017 (6)	0.0005 (6)
C5	0.0406 (8)	0.0291 (7)	0.0295 (7)	0.0003 (6)	−0.0025 (6)	0.0030 (5)
N2	0.0554 (9)	0.0296 (7)	0.0386 (7)	0.0022 (7)	0.0041 (6)	0.0022 (5)
C6	0.0463 (9)	0.0349 (8)	0.0288 (7)	−0.0010 (7)	−0.0045 (6)	0.0028 (6)
N1	0.0443 (8)	0.0340 (7)	0.0315 (6)	−0.0034 (6)	0.0023 (5)	−0.0034 (5)

### Geometric parameters (Å, °)

**Table:**

O1—C7	1.203 (2)	O2—H2	0.91 (3)
C3—N2	1.313 (2)	C5—C6	1.392 (2)
C3—C4	1.395 (2)	C5—H5	0.9300
C3—H3	0.9300	N2—N1	1.334 (2)
C4—C5	1.367 (2)	C6—N1	1.308 (2)
C4—C7	1.497 (2)	C6—H6	0.9300
O2—C7	1.309 (2)	N1—H1	0.92 (3)
			
N2—C3—C4	123.60 (15)	C4—C5—C6	117.17 (16)
N2—C3—H3	118.2	C4—C5—H5	121.4
C4—C3—H3	118.2	C6—C5—H5	121.4
C5—C4—C3	118.55 (15)	C3—N2—N1	115.32 (14)
C5—C4—C7	122.31 (15)	N1—C6—C5	119.27 (15)
C3—C4—C7	119.12 (14)	N1—C6—H6	120.4
C7—O2—H2	111.4 (17)	C5—C6—H6	120.4
O1—C7—O2	126.14 (16)	C6—N1—N2	126.06 (15)
O1—C7—C4	121.39 (16)	C6—N1—H1	120.2 (16)
O2—C7—C4	112.47 (13)	N2—N1—H1	113.5 (16)

### Hydrogen-bond geometry (Å, °)

**Table:**

*D*—H···*A*	*D*—H	H···*A*	*D*···*A*	*D*—H···*A*
O2—H2···Cl1	0.91 (3)	2.05 (3)	2.9464 (14)	169 (2)
N1—H1···Cl1^i^	0.92 (3)	2.15 (3)	3.0373 (15)	160 (2)

Symmetry codes: (i) *x*+1/2, −*y*+3/2, *z*+1/2.
